# Activation of Hypoxia-Inducible Factor-1α Signaling Pathway Has the Protective Effect of Intervertebral Disc Degeneration

**DOI:** 10.3390/ijms222111355

**Published:** 2021-10-21

**Authors:** Jin-Woo Kim, Hyun-Ju An, HyunJeong Yeo, Yunhui Jeong, HyeonHae Lee, Jusung Lee, Kisik Nam, Jongheon Lee, Dong-Eun Shin, Soonchul Lee

**Affiliations:** 1 Department of Orthopaedic Surgery, Nowon Eulji Medical Center, Eulji University, Seoul 01830, Korea; jinwu3911@hanmail.net (J.-W.K.); woal222@hanmail.net (J.L.); 2 Department of Orthopaedic Surgery, CHA Bundang Medical Center, CHA University, 59 Yatap-ro, Bundang-gu, Seongnam-si 13496, Gyeonggi-do, Korea; yks486ahj@naver.com (H.-J.A.); lallalla106@naver.com (H.Y.); jeongyunhui92@gmail.com (Y.J.); aotcnlsl@gmail.com (H.L.); medisup8684@gmail.com (J.L.); keysix777@naver.com (K.N.)

**Keywords:** intervertebral disc, degeneration, hypoxia inducible factor-1α, protection

## Abstract

Intervertebral discs (IVDs) have poor nutrient diffusion, because the nucleus pulposus (NP) lacks direct vascular supply and likely generates adenosine triphosphate by anaerobic glycolysis. Regulation of glycolysis is mediated by hypoxia-inducible factor-1α (HIF-1α), a transcription factor that responds to local oxygen tension. Constitutively active HIF-1α (CA HIF-1α) was created by point mutation and determined the protective role of HIF-1α in IVD degeneration. Under fluoroscopy, rat caudal IVD segments were stabbed by a needle puncture, and pcDNA3- HIF-1α wild-type (WT) or pcDNA3-CA HIF-1α was transfected into NP cell lines. The constitutive activity of CA HIF-1α was analyzed using a luciferase assay after cell lysis. Next, IVD tissue samples were retrieved from five patients with degenerative lumbar spinal stenosis at the time of surgery, and NP cells were cultured. NP cells were transfected with CA HIF-1α, and relevant gene expression was measured. HIF-1α protein levels in the nucleus were significantly higher, and transcriptional activity was 10.3-fold higher in NP cells with CA HIF-1α than in those with HIF-1α WT. Gene transfer of CA HIF-1α into NP cells enhanced the expression of Glut-1, Glut-3, aggrecan, type II collagen, and Sox9. Moreover, CA HIF-1α reduced the apoptosis of NP cells induced by the Fas ligand. The HIF-1α and collagen 2 expression levels were notably increased in the NP cells of the CA HIF-1α transfected segments in histology and immunohistochemistry study. Collectively, these results suggest that activation of HIF-1α signaling pathway may play a protective role against IVD degeneration and could be used as a future therapeutic agent.

## 1. Introduction

Progressive reduction in proteoglycan content in the nucleus pulposus (NP) that deteriorates with age can lead to intervertebral disc (IVD) degeneration [1]. IVD degeneration is one of the predominant causes of lower back pain [2]. Multiple factors, including altered mechanical loading, reduced nutrient supply, hereditary factors, and changes in the extracellular microenvironment with age, have been implicated in the initiation and progression of the degenerative cascade [3,4,5,6]. However, the mechanism underlying IVD degeneration has not been clearly identified.

Cell density associated with fully developed, healthy IVD is very low, and the poor diffusion of nutrients and accumulation of metabolic waste products, such as lactate, present IVD cells with further environmental challenges due to the absence of microvasculature [7,8,9]. That is, the NP has no direct vascular supply, and adenosine triphosphate (ATP) is likely generated by anaerobic glycolysis [10]. Regulation of glycolysis is mediated by hypoxia-inducible factor-1α (HIF-1α), a transcription factor that responds to the local oxygen tension, and it has been shown to be expressed by NP cells [11].

HIF-1α promotes the transcription of key glycolytic enzymes, allowing cells to adapt to low oxygen tension and to inhibit ATP generation through oxidative phosphorylation [12]. The activity of HIF-1α within a cell is oxygen-sensitive, decreases in response to normoxia, and increases under hypoxic conditions. Under normoxic conditions, HIF-1α is hydroxylated at two specific proline residues (Pro402 and Pro564) in the oxygen-dependent degradation domain by the prolyl 4-hydroxylase domain-containing (PHD) enzyme, which leads to polyubiquitination and proteasomal degradation of HIF-1α by the von Hippel–Lindau tumor suppressor (VHL) protein, a component of E3 ubiquitin-protein ligase [13,14]. Under hypoxic conditions, inhibition of PHD enzyme activity spares HIF-1α from polyubiquitination and proteasomal degradation, thereby allowing HIF-1α to accumulate and to translocate to the nucleus, where it dimerizes with HIF-1β and binds to the hypoxia-responsive element (HRE) sequences of target gene promoters. In the nucleus, the transcriptional activity of the HIF-1α heterodimer is regulated by the hydroxylase factor inhibiting HIF-1 (FIH-1) [15]. The FIH-1 enzyme hydroxylates Asn803 on HIF-1α, which represses HIF-1α transactivation by preventing the transcriptional coactivator p300/CBP from binding to the HIF-1α C-transactivation domains (C-TADs) [16,17].

Recently, several researchers have reported that the PHD/HIF-1 axis is related to IVD degeneration [18,19]. However, limited information is available regarding the role of HIF-1α in IVD degeneration, despite the well-known effects of oxygen tension on HIF-1α.

To investigate the role of HIF-1α in IVD degeneration, the availability of an experimental animal model may be essential. A recently developed needle-puncture model using a stab incision (also called “anulotomy”) at the IVD to induce degeneration was used for its convenience, minimal invasiveness, and cost-effectiveness [20,21]. This procedure can produce morphological and biochemical alterations similar to those observed with human IVD degeneration. With lower costs and less precision needed, the caudal discs of a rat model using needle puncture could be an effective way to demonstrate the morphological changes associated with IVD degeneration.

In this study, we hypothesized that constitutively active HIF-1α (CA HIF-1α) has a protective role against IVD degeneration. To confirm this hypothesis, we constructed CA HIF-1α, which resisted degradation even under normoxic conditions, by point mutation, and the effect of CA HIF-1α on IVD degeneration was investigated by analyzing glycolysis, apoptosis, and vascular endothelial growth factor (VEGF) and matrix gene expression. We performed magnetic resonance imaging (MRI) and histological and immunohistochemical assessments in a rat model, which demonstrated the effect of CA HIF-1α on IVD degeneration.

## 2. Results

### 2.1. Hypoxia Increases Aggrecan and Collagen II mRNA Expression in Nucleus Pulposus (NP) Cells

**Figure 1:**
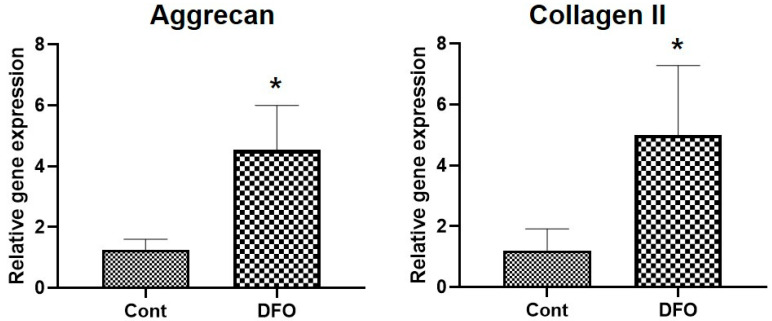
Hypoxia increases mRNA expression of Aggrecan and Collagen II in nucleus pulposus (NP) cells. The mRNA levels of Aggrecan and Collagen II in NP cells treated with DMSO or Deferoxamine (150 µM) were analyzed by qRT–PCR. β-Actin was used as a normalizer. Error bars represent mean ± SD. * *p* < 0.05 (n = 6).

To assess the effect of HIF-1α on disc regeneration, NP cells were cultured in either DMSO or hypoxia-mimetic agents desferrioxamine (DFO) for 48 h, and the mRNA level was quantified using qRT-PCR. NP cells cultured under hypoxia showed more Aggrecan and Collagen II mRNA expression than those cultured in normoxia (Figure 1).

### 2.2. Robust Transcriptional Activity of pHIF-1α Mut

**Figure 2:**
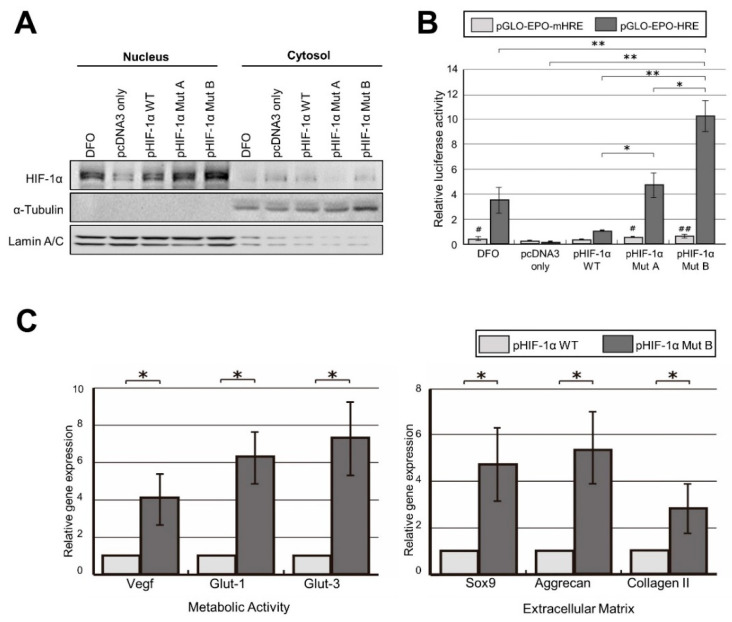
CA HIF-1α (pHIF-1α Mut B) enhances HIF-1α transcriptional activity. (**A**) pHIF-1α Mut A and B showed increased protein levels under normoxic condition. DFO only, pcDNA3 only, pHIF-1α WT, pHIF-1αMut A, and pHIF-1α Mut B were transfected into NP cells. After 2 days, nuclear and cytoplasmic fractions were obtained, and the HIF-1α protein levels were examined by Western blotting. A pHIF-1α Mut A and B had significantly higher HIF-1α protein levels than pHIF-1α WT in both nuclear and cytoplasmic fractions. DFO was used as a positive control. Lamin A/C was used as nuclear control. α-Tubulin was used as nuclear control. (**B**) CA HIF-1α (pHIF-1α Mut B) enhances HIF-1α transcriptional activity. A pHIF-1α Mut A and B have higher transcriptional activity specifically related to the HRE sequence. DFO only, pcDNA3 only, pHIF-1α WT, pHIF-1α Mut A, and pHIF-1α Mut B were transfected into NP cells with pGL2-EPO-HRE or pGL2-EPO-mHRE under normoxic condition. After 2 days, cells were lysed, and luciferase activities measured. Both mutants of HIF-1α showed increased transcriptional activity specifically related to the EPO-HRE sequence. A pHIF-1α Mut B was more active than Mut A. Firefly luciferase activity was normalized to that of Renilla luciferase, which was used as an internal control. * and ** mean *p*-value < 0.05 and <0.01 comparison within the pGL2-EPO-HRE groups, respectively. # and ## mean *p*-value < 0.05 and <0.01, respectively, between pGL2-EPO-HRE and pGL2-EPO-mHRE in the same group. (**C**) CA HIF-1α (pHIF-1α Mut B) upregulates the genes related to the metabolic activity and extracellular matrix production. NP cells from patient tissues were treated with control pcDNA3 or CA HIF-1α. After 48 h, qRT-PCR analysis was performed. (Left) CA HIF-1α induced significant upregulation of VEGF, Glut-1, and Glut-3 expression in NP cells. (Right) CA HIF-1α increased Sox9, aggrecan, and type II collagen expression in NP cells with the statistical significance. The total mRNA quantity was normalized to that of GAPDH. * *p*-value < 0.05.

To determine whether pHIF-1α Mut A and B were degraded under normoxic conditions, nuclear and cytoplasmic fractions were obtained, and the HIF-1α protein levels of each group were analyzed by Western blotting. HIF-1α protein levels in both pHIF-1α Mut A and B were significantly higher than those of pHIF-1α WT in the nuclear fractions (*p* < 0.01) (Figure 2A). This suggests that HIF-1α mutated cells have increased stability and nuclear localization even under normoxic conditions, as HIF-1α is not degraded by the ubiquitin–proteasome system.

Next, to evaluate the transcriptional activity of pHIF-1α Mut A and B, a luciferase reporter assay was performed. In the luciferase reporter assay performed with pGL2-EPO-HRE reporter plasmids, pHIF-1α Mut A and B showed 4.7- and 10.3-fold higher transcriptional activity, respectively, compared with pHIF-1α WT with statistical significance, while no significant difference between the transcriptional activity of pHIF-1α Mut A and B and that of pHIF-1α WT was observed in the assay performed with pGL2-EPO-mHRE (Figure 2B). Therefore, the mutants of HIF-1α showed increased transcriptional activity in a specific manner in the EPO-HRE sequence. pHIF-1α Mut B was selected for further study because pHIF-1α Mut B was more active than pHIF-1α Mut A. A pHIF-1α Mut B will be referred to as CA HIF-1α from this point.

Then, to investigate the effects of CA HIF-1α (pHIF-1α Mut B) on VEGF and glucose transporter (Glut-1 and Glut-3) expression, NP cells from patient tissues were transfected with pcDNA3, pHIF-1α WT, and CA HIF-1α. After 48 h, qRT-PCR analysis was performed to evaluate VEGF, Glut-1, and Glut-3 expression. CA HIF-1α induced significant upregulation of VEGF, Glut-1, and Glut-3 expression in NP cells. To validate the effect of CA HIF-1α on the disc matrix, Sox9, aggrecan, and type II collagen expression were also analyzed, and the results indicated that CA HIF-1α increased Sox9, aggrecan, and type II collagen expression in NP cells (Figure 2C).

### 2.3. CA HIF-1α Decreased the Apoptosis of NP Cells

**Figure 3:**
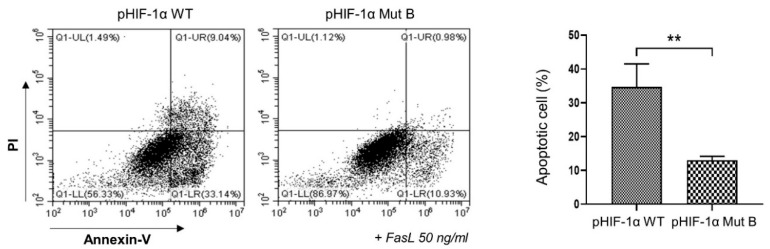
CA HIF-1α (pHIF-1α Mut B) reduces the apoptosis induced by FasL treatment. NP cells were transfected with pHIF-1α WT or CA HIF-1α, and NP cell apoptosis was induced by the treatment with 50 ng/mL of FasL. After 24 h, annexin V-positive cells were enumerated by flow cytometry. PI, propidium iodidin ** *p*-value < 0.01.

To examine the role of CA HIF-1α in NP cell apoptosis, NP cells were transfected with pHIF-1α WT or CA HIF-1α, and apoptosis was induced by treatment with 50 ng/mL FasL. After 24 h, apoptosis was measured by enumerating annexin V-positive cells. FasL induced apoptosis in 34.7% of NP cells, although CA HIF-1α reduced the proportion of apoptotic cells to 12.5% (Figure 3). This result implies that CA HIF-1α protects NP cells from apoptosis during IVD degeneration.

### 2.4. Radiographic and MRI Assessment, and Histological Evaluation of CA HIF-1α

**Figure 4:**
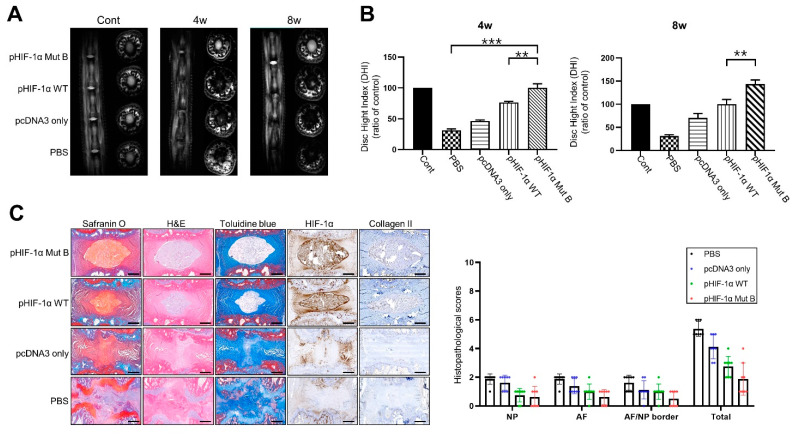
CA HIF-1α (pHIF-1α Mut B) ameliorates disc degeneration. (**A**) Representative T2 weighted micromagnetic resonance imaging (MRI) of rat tail in the four groups at 4 and 8 weeks. (**B**) Disc height index (DHI) of four groups at 4 and 8 weeks. ** *p*-value < 0.01, *** *p*-value < 0.005. (**C**) Histology and immunohistochemistry in puncture-induced rat IVD degeneration model. Histological analysis of intervertebral discs by Safranin O, H&E, Toluidine blue staining, and immunohistochemical staining of HIF-1α and collagen II in the nucleus pulposus (NP) in all groups. Scale bars represent 100 μm. ** *p*-value < 0.01, *** *p*-value < 0.005.

The T2 signal intensity was decreased in the PBS- and pcDNA3-alone segments at 4 and 8 weeks following needle puncture. However, the signal intensity in the pHIF-1α WT and pHIF-1α Mut B segments was increased at 4 and 8 weeks following injection compared with that of the PBS and pcDNA3 alone segments, and the difference was statistically significant (*p* < 0.05; Figure 4A). The degree of disc height index (DHI) reduction comparing control in the pHIF-1α WT and pHIF-1α Mut B segments was smaller than that in the PBS and pcDNA3 segments at 4 and 8 weeks after injection; this difference was also statistically significant (*p* < 0.05; Figure 4B).

Representative images of safranin O staining are shown in Figure 4C. The NP area in the disc stained strongly for safranin O, although the stained levels were not homogenous in the PBS and pcDNA3 segments. Histological analysis of IVD tissues was performed using H&E and toluidine blue staining. In the PBS and pcDNA3 segments, ruptured and serpentine-patterned fibers were observed in the AF. Furthermore, the border between the AF and NP was interrupted and unclear and exhibited features of severe degeneration. However, the AF and the border between the AF and NP in the pHIF-1α WT and pHIF-1α Mut B segments demonstrated an intact circumferential AF, and the border between the AF and NF was clear compared with those observed in the PBS and pcDNA3 segments. Immunohistochemical staining revealed that the staining for HIF-1α and collagen 2 were stronger in the pHIF-1α Mut B transfected segments compared with pHIF-1α WT segments. HIF-1α and collagen 2 expression levels were negligible in the PBS segments. Conversely, the HIF-1α and collagen 2 expression levels were notably increased in the NP cells of the pHIF-1α Mut B transfected segments. A histopathology scoring system was applied according to Lai et al. [22], and pHIF-1α Mut B had a significantly lower score than the other segments.

## 3. Discussion

In this study, mutants of HIF-1α were constructed by replacing Pro402, Pro564, and Asn803 with alanines to investigate the protective role of HIF-1α in IVD degeneration. HIF-1α was first identified as a critical factor for the inducible activity of the EPO 3′ enhancer and is now recognized to be a key regulator of gene expression in response to changes in cellular oxygen tension [23]. The N-terminal half of HIF-1α contains basic helix-loop-helix and Per-aryl hydrocarbon receptor nuclear translocator-Sim, which are required for dimerization and DNA binding [24]. The C-terminal half contains domains required for degradation and transactivation, the oxygen-dependent degradation domain, which confers oxygen-dependent instability, two independent TADs, and an inhibitory domain that negatively regulates TAD [25]. In addition, we demonstrated that pHIF-1α Mut B injection significantly alleviated IVD degeneration using a needle puncture model in rats, as determined by MRI and histological and immunohistochemical analyses. 

Our results demonstrated that CA HIF-1α had a significantly increased protein level and nuclear localization when transfected into NP cells. This result indicates that CA HIF-1α is not degraded under normoxic conditions and indeed is constitutively active. Moreover, CA HIF-1α transactivates reporter gene expression specifically in the EPO-HRE sequence. Therefore, these results demonstrate that CA HIF-1α can also be constructed by point mutation, not by deletion as reported previously, and may be a useful tool for further investigating the biological roles of HIF-1α under normoxic conditions [26,27].

In NP cells transfected with CA HIF-1α, the expression of Glut-1 and Glut-3, glucose transporters in NP cells, was significantly enhanced under normoxic conditions. This suggests that CA HIF-1α facilitates energy metabolism in NP cells by stimulating anaerobic glycolysis. Degeneration is characterized by increased degradation of the normal IVD matrix by locally produced matrix metalloproteinases and a disintegrin and metalloproteinase with thrombospondin motifs [28]. In addition, the matrix produced in degenerated IVDs differs from that in normal IVDs, as a consequence of switches in the production of collagen within the inner annulus fibrosus and NP from type II to type I, and in the synthesis of proteoglycan from aggrecan to versican, biglycan, and decorin [29,30]. The resultant changes within the extracellular matrix have several consequences, including loss of structural integrity, decreased hydration, and reduced ability to withstand load.

Histologically, in young adults, NP contains a high percentage of type II collagen synthesized by disc cells. With aging, the synthesis of type II collagen declines, whereas the degradation of existing type II collagen gradually increases [31,32]. Furthermore, type I collagen production within the disc increases, leading to a higher proportion of less compliant type I collagen [33].

The decline in type II collagen within NP features prominently in the pathogenesis of IVD degeneration, and reversing this trend may provide an opportunity to modify early IVD degeneration. The regulation of type II collagen synthesis undoubtedly involves a complex interplay between local and systemic factors. Recent studies have demonstrated that Sox9 is an essential transcription factor for type II collagen synthesis and chondrogenesis [34,35,36]. Aggrecan and type II collagen, major components of proteoglycans and the extracellular matrix of NP cells, play a role in spinal stability. Sox9 is a representative chondrogenesis-inducing transcription factor. In this study, Sox9, aggrecan, and type II collagen expression increased in NP cells transfected with CA HIF-1α. This suggests that transfer of the CA HIF-1α gene into NP cells may stimulate extracellular matrix synthesis and play a protective role in IVD degeneration, because enhanced matrix degradation is a hallmark of IVD degeneration.

In degenerative IVD tissues, increased cell apoptosis and high expression of both Fas and FasL have been reported [37,38]. In this study, apoptosis of NP cells was induced by treatment with FasL, and apoptosis was markedly reduced in NP cells transfected with HIF-1α. Therefore, CA HIF-1α gene transfer into NP cells may decrease apoptosis and protect against IVD degeneration.

We have, for the first time, identified an association between IDD and the CA HIF-1α, especially pHIF-1α Mut B. Sutter et al. demonstrated that missense mutations increase HIF-1α expression under normoxic conditions by blocking ubiquitination and missense mutations and/or deletions involving several different regions of HIF-1α, resulting in constitutive activation and transcriptional activity in normoxic cells.

We utilized MRI technology to assess IVD degeneration intravitally and continuously in vivo. The signal intensity of the T2-weighted images and disc height on the MR scans showed that the water content and NP volume in the IVD as well as the loss of water content and NP volume were associated with a decrease in the T2-weighted signal intensity and disc height. Therefore, IVD regeneration results in an increase in T2-weighted signal intensity and disc height. Our results demonstrated that degenerative changes in NP induced by needle puncture were recovered following pHIF-1α Mut injection, especially in pHIF-1α Mut B. The T2-weighted signal intensity and disc height of the pHIF-1α Mut B were significantly higher than those of the other groups at 4 and 8 weeks after injection. 

The degenerated disc was less elastic, consequently prohibiting the ability to absorb and to endure spinal loading [16]. Type II collagen in IVD was demonstrated to be present at normal levels in the NP and in the inner layer of the AF; however, type I collagen was found to be present at normal levels in the AF and degenerative NP [39]. In a rabbit annular stab-injury model, aggrecan and type IIa collagen mRNA levels were decreased and could not be restored to normal levels, whereas type Ia collagen mRNA levels gradually increased throughout the course of degeneration [20]. We recognized that this rat model has limitation, because we used the multiple discs within the same animal. Despite this, this study demonstrated that in degenerative discs injected with pHIF-1α Mut B, VEGF, aggrecan, and collagen II levels in the NP were restored. Our results indicate that CA HIF-1α may be an optimal method for maintaining the normal extracellular matrix components and mechanical properties of discs.

In summary, we successfully constructed HIF-1α mutant form, which was not degraded under normoxic conditions by point mutation. CA HIF-1α showed approximately 10.3-fold higher transcriptional activity than HIF-1α WT from the EPO-HRE reporter plasmid. Gene transfer of CA HIF-1α into NP cells using a recombinant adenovirus enhances the expression of glycolysis-involved genes and matrix synthesis-related genes. Furthermore, CA HIF-1α reduced apoptosis of NP cells. Using MRI, histological, and immunohistochemical analyses, we found that CA HIF-1α inhibited IVD degeneration and maintained NP in the disc of rat model.

Collectively, these results suggest that activation of the HIF-1α signaling pathway may play a protective role against IVD degeneration and could be used as a future therapeutic agent.

## 4. Materials and Methods

### 4.1. Construction of Mutant HIF-1α 

pcDNA3 plasmid and pcDNA3 HIF-1α wild-type (pHIF-1α WT, plasmid#18949) were purchased from Invitrogen and Addgene, Inc., respectively. The HIF-1α wild-type (HIF-1α WT) gene was mutated using a polymerase chain reaction-based (PCR-based) mutagenesis method (Stratagene, Lajolla, CA, USA) according to the manufacturer’s instructions. Two types of HIF-1α mutants (HIF-1α Mut) were created by point mutation as follows: (1) pHIF-1α Mut A was created by replacing proline 402 (Pro402) and Pro564 with alanines, and (2) pHIF-1α Mut B was created by replacing Pro402, Pro564, and asparagine 803 (Asn803) with alanines. These three sites were selected because Pro402 and Pro564 were involved in oxygen-dependent degradation, and mutations at Asn803 are known to prevent the interaction with p300 for transcriptional activation [40].

### 4.2. Validation of HIF-1α Mut by Western Blot Analysis

To compare the activity of HIF-1α WT proteins between mutants A and B and the control under normoxic conditions (20% O_2_), the following five groups were transfected into NP cells using Lipofectamine (Invitrogen, Waltham, MA, USA): (1) Desferrioxamine (DFO) only, (2) pcDNA3 only, (3) pHIF-1α WT, (4) pHIF-1α Mut A, and (5) pHIF-1α Mut B. DFO was used as a positive control, because it inhibits both PHD enzyme and FIH-1 enzyme activities [41]. The transfected cells were lysed using a cell fractionation kit (Cell Signaling Technology, Danvers, MA, USA). The cells were separated into nuclear and cytoplasmic fractions in Nupage 4–12% Bis-Tris gels (Invitrogen, Waltham, MA, USA) and then transferred to polyvinylidene difluoride membranes (Millipore, Billerica, MA, USA). The membranes were blocked with 5% skim milk and washed with phosphate-buffered saline and 0.2% Tween 20 buffer. The membranes were blotted with mouse anti-HIF-1α (Cell Signaling Technology, Danvers, MA, USA), mouse anti-nuclear lamin A/C, or anti-α-tubulin (Santa Cruz Biotechnology, Dallas, TX, USA) antibodies. The blots were treated with goat anti-mouse conjugated with horseradish peroxidase (Santa Cruz Biotechnology, Dallas, TX, USA) and visualized using an enhanced chemiluminescence system (Uppsala, Sweden).

### 4.3. Validation of pHIF-1α Mut by Luciferase Reporter Assay

To determine the transcriptional activity of pHIF-1α Mut A and B, a luciferase reporter assay was performed. The following two different types of luciferase reporter plasmids were used to determine whether pHIF-1α Mut A and B had increased transcriptional activity specifically related to the erythropoietin (EPO)-HRE sequence: (1) pGL2-EPO-HRE, containing the wild-type HRE sequence in the EPO gene, and (2) pGL2-EPO-mHRE, containing the mutant HRE sequence in the EPO gene. DFO only, pcDNA3 only, pHIF-1α WT, and pHIF-1α Mut A and B were co-transfected into HEK NP cells with pGL2-EPO-HRE or pGL2-EPO-mHRE using Lipofectamine (Invitrogen, Waltham, MA, USA). The cells were lysed, and luciferase assays (Promega, Madison, WI, USA) were performed using a luminometer (Titertek Berthold FB14, Bad Wildbad, Germany). Firefly luciferase activity was normalized to that of Renilla luciferase, which was used as an internal control.

### 4.4. Patients and Tissue Samples

The Institutional Review Board of the CHA Bundang Medical Center approved this study. Patients diagnosed with degenerative lumbar spinal stenosis were recruited after obtaining informed consent. Before sample collection, the degree of IVD degeneration was evaluated according to the Pfirrman grading system [42], and degenerative discs of grade IV or higher were collected in this study.

### 4.5. Human NP Cell Isolation and Cultures

Human NP cells donated by patients were obtained within 2 h after surgery. NP tissues were identified and separated by a stereotaxic microscope. The NP tissues were washed with phosphate-buffered saline (PBS) and digested for 40 min in 0.2% pronase (Gibco BRL, Carlsbad, CA, USA). Following being washed, the tissues were incubated in 0.25% type II collagenase (Gibco BRL, Carlsbad, CA, USA) at 37 °C under gentle agitation for 4 h. The cell suspension was centrifuged at 300× *g* for 10 min, and the pellet was washed with Dulbecco’s modified Eagle’s medium (DMEM) (Invitrogen, Waltham, MA, USA). The cells were plated in culture dishes and maintained in DMEM supplemented with 10% fetal bovine serum and 50 µg/mL gentamicin (Invitrogen, Waltham, MA, USA) at 37 °C in 5% CO_2_. First-passage cells maintained in a monolayer were obtained, and second-passage cells were used in the experiments. NP cells were transfected with empty pcDNA3 only or pHIF-1α (WT, Mut A and Mut B).

### 4.6. Quantitative Real-Time Polymerase Chain Reaction (qRT-PCR)

Total RNA was isolated from NP cells using TRIzol reagent (Invitrogen, Waltham, MA, USA). First-strand cDNA was synthesized using the SuperScript system (Invitrogen, Waltham, MA, USA). PCR amplification conditions were as follows: denaturation at 94 °C for 20 s, annealing at 55 °C for 30 s, and extension at 72 °C for all genes. PCR reactions were performed using the QuantiTech SYBR Green PCR kit (Qiagen, Valencia, CA, USA) and an Exicycler 96 system (Bioneer, Daejeon, Korea). Specific primers for the genes examined were based on their PrimerBank sequences and are listed in Table 1.

### 4.7. Preparation of Subcellular Fractions

NP cells were cultured and fractionated into cytosolic and nuclear fractions using a cell fractionation kit (Abcam, Inc., Cambridge, UK) according to the manufacturer’s instructions. Trypsinized NP cells were collected and centrifuged for 5 min at 300× *g*. The collected cells were washed with buffer A (wash buffer). Then, the cells were resuspended in buffer A. An equal volume of buffer B (lysis buffer for Cyt extraction) was added to the cell suspensions, mixed via pipetting, and then incubated for 7 min at room temperature (RT). The cell lysates were centrifuged at 5000× *g* for 1 min at 4 °C, and the cytosol fractions (cell lysate supernatants) were collected in new tubes. The remaining pellets containing the nuclear protein fraction were resuspended in buffer A.

### 4.8. Western Blotting

Transfected NP cell lysates were boiled in an SDS sample buffer, resolved by SDS-PAGE, and transferred to nitrocellulose membranes. After the transfer, the membranes were blocked in 5% skim milk in TBST (10 mM Tris–HCl, pH 8.0, 150 mM NaCl, 0.05% Tween 20) for 40 min and incubated with the specific primary antibodies in the blocking solution for overnight at 4 °C. Antibodies against HIF-1α (sc-13515, 1:1000) and α-Tubulin (sc-8035, 1:1000) were purchased from Santa Cruz Biotechnology (Dallas, TX, USA). Antibodies against Lamin A/C (#4777, 1:1000) was purchased from Cell Signaling Technology (Danvers, MA, USA). The membranes were then washed with TBST and incubated with horseradish peroxidase (HRP)-conjugated secondary antibody followed by detection using an enhanced chemiluminescence system (Amersham Pharmacia Biotech, Piscataway, NJ, USA).

### 4.9. Apoptosis Assay

NP cells were transfected with pHIF-1α WT or CA-HIF-1α and then treated with 50 ng/mL of Fas ligand (FasL) (R&D Systems, Minneapolis, MN, USA). After 24 h, apoptosis was measured using an apoptosis detection kit (Miltenyi Biotec, Bergisch Gladbach, Germany) according to the manufacturer’s instructions. Briefly, cells were collected and incubated with fluorescein isothiocyanate-labeled annexin (V annexin V-FITC) in Annexin V Binding Buffer. Annexin V-positive cells were enumerated by flow cytometry (FACScan™ Calibur, Becton Dickinson, San Jose, CA, USA).

### 4.10. Preparation of Animals

Ten male Sprague Dawley rats (each weighing 220–250 g and 2 months old) were used in this study. The animals were housed with free access to commercial rodent chow and water. The temperature was maintained at 24 °C, and the light schedule comprised 12 h of daylight starting at 8:00 a.m. and 12 h of darkness starting at 8:00 p.m. Humidity was maintained at 50%. All experiments were performed according to the guidelines for the ethical treatment of animals approved by the Laboratory Animal Ethics Committee of CHA Bundang Medical Center (Seongnam, Korea).

### 4.11. Surgical Procedure

**Figure 5:**
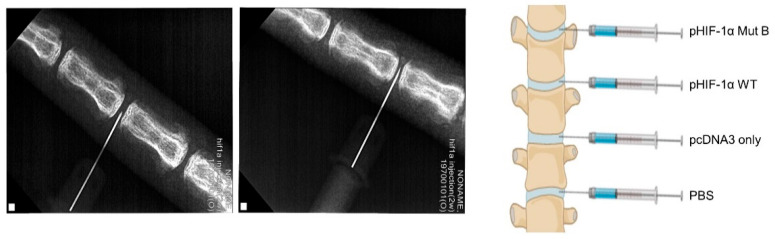
Schematic demonstrating the surgical model. (**Left**) Under X-ray, rat caudal IVDs were stabbed by a needle at a depth of 5 mm from the subcutaneous surface to the NP. (**Right**) Schematic illustration demonstrating transfection sites of rat caudal IVDs.

The rat tail segments were divided into four groups: (1) phosphate-buffered saline (PBS), (2) pcDNA3 only, (3) pHIF-1α WT, and (4) pHIF-1α Mut B. Ten rat tails were prepared for the drug treatment. The animals were anesthetized by inhalation of anesthetic isoflurane, and the operative field was prepared in a sterile fashion. After identifying the level of the caudal segments by fluoroscopy, a puncture was made parallel to the endplates through the annulus fibrosus (AF) into the NP using a syringe needle (21-gauge) (Figure 5) [43]. The needle was then rotated by 180° and held for 5 s. During needle puncture, fluoroscopy was performed to ensure that the needle reached the center of the NP [20]. For the MRI assessment, the caudal segments 4/5 and 5/6 from the same rat tail were used for the PBS- and pcDNA3-only treatment, and the caudal segments 6/7 and 7/8 for the pHIF-1α WT and pHIF-1α Mut B (Figure 5).

### 4.12. Radiographic and MRI Evaluation

Images were obtained using a 3.0T MRI machine (Philips Healthcare, Amsterdam, The Netherlands) using a dedicated coil for small animals. The animals were serially followed by MRI immediately and 4 and 8 weeks following injection. At each time point, the rats were anesthetized with a 2% isoflurane/oxygen mixture throughout the MRI examination. The animals were placed in a prone position, and their tails were straightened in the MR scanner. Serial T2-weighted sagittal and transverse images covering the entire experimental disc area were obtained, and T2-weighted signal intensity and disc height were measured using Image J software (National Institutes of Health, Bethesda, MD, USA). Additionally, intervertebral disc height index (DHI) under X-ray immediately before and after injection was calculated using modification of previous methods [44,45,46]. Image assessments were conducted by two independent, blinded, and experienced observers. The data are presented as the means of the two evaluations.

### 4.13. Histological Analysis

At 8 weeks after injection, all rats were killed, and the discs were harvested for histological examination. Each disc was fixed, decalcified, using 10% ethylenediaminetetraacetic acid (EDTA; pH = 7.4) for 1 month. The EDTA solution was replaced every 2 days. The decalcified discs were washed, dehydrated and then processed for paraffin embedding and sectioning into sagittal sections (5 μm thickness) using a microtome. Sagittal sections were stained with hematoxylin and eosin (H&E), safranin O, and toluidine blue. Histological images were analyzed qualitatively under a light microscope (BX51, Olympus, Inc., Tokyo, Japan) at magnifications ranging from 4 to 100× to investigate changes in the NP, AF, and endplates. The extent of disc degeneration was graded using a height index as described previously by Masuda et al. [47].

### 4.14. Immunohistochemical Staining

Immunohistochemistry (IHC) was performed using a Ready-to-use IHC/ICC kit (BioVision, Inc., Milpitas, CA, USA) according to the manufacturer’s protocol. Briefly, paraffin-embedded sections were deparaffinized, rehydrated, immersed in a retrieval solution (10 mmol/L citrate, pH 6.0), and then placed in a microwave for 10 min. Endogenous peroxidase activity was blocked using 3% hydrogen peroxide for 10 min. The slides were incubated in 3% H2O2 at room temperature for 30 min to quench endogenous peroxidase activity and then blocked in blocking buffer (BioVision, Inc., Milpitas, CA, USA) at room temperature for 10 min, followed by incubation with anti- HIF-1α (sc-13515; 1:100) or anti-collagen II (1:100) antibodies at room temperature for 30 min. After incubation with HRP-anti-mouse or -rabbit IgG polymer at room temperature for 20 min and washing with PBS, the tissue sections were treated with 3,3′-diaminobenzidine at room temperature for 10 min, followed by counterstaining with hematoxylin at room temperature for 1 min.

### 4.15. Statistical Analyses

All data were analyzed at least three times for each group. The means of the groups were compared using the Mann–Whitney U test or Kruskal–Wallis test with post hoc Bonferroni tests. Means and standard deviations were calculated from numerical data, as presented in the text, figures, and figure legends. In the figures, bar graphs represent the means, whereas error bars represent one standard deviation. SPSS version 18.0 (SPSS, Chicago, IL, USA) was used for all statistical analyses. The Kruskal–Wallis test was used to analyze the differences in T2 signal intensities, and one-way analysis of variance was used to analyze the differences in disc height. Statistical significance was set at *p* < 0.05.

## Figures and Tables

**Table 1:** qRT-PCR genes and primer sequence.

Gene Name	Forward (5′–3′)	Reverse (5′–3′)
GAPDH	TCCCTGAGCTGAACGGGAAG	GGAGGAGTGGGTGTCGCTGT
VEGF	CCATGAACTTTCTGCTGTCTT	TCGATCGTTCTGTATCAGTCT
Glut-1	CTTTGTGGCCTTCTTTGAAGT	CCACACAGTTGCTCCACAT
Glut-3	AGGCTCGATGCTGTTCATCT	ACCGGCTTCCTCATTACCTT
SOX9	GCGGAGGAAGTCGGTGAAGA	CCCTCTCGCTTCAGGTCAGC
Aggrecan	CACTGTTACCGCCACTTCCC	ACCAGCGGAAGTCCCCTTCG
Type II collagen	CTATCTGGACGAAGCAGCTGGCA	ATGGGTGCAATGTCAATGATGG

## Data Availability

Not applicable.

## References

[1] Richardson S.M., Mobasheri A., Freemont A.J., Hoyland J.A. (2007). Intervertebral disc biology, degeneration and novel tissue engineering and regenerative medicine therapies. Histol. Histopathol..

[2] Peterson C.K., Bolton J.E., Wood A.R. (2000). A cross-sectional study correlating lumbar spine degeneration with disability and pain. Spine.

[3] Stokes I.A., Iatridis J.C. (2004). Mechanical conditions that accelerate intervertebral disc degeneration: Overload versus immobilization. Spine.

[4] Battié M.C., Videman T. (2006). Lumbar disc degeneration: Epidemiology and genetics. J. Bone Jt. Surg. Am..

[5] Urban J.P., Smith S., Fairbank J.C. (2004). Nutrition of the intervertebral disc. Spine.

[6] Zhao C.Q., Wang L.M., Jiang L.S., Dai L.Y. (2007). The cell biology of intervertebral disc aging and degeneration. Ageing Res. Rev..

[7] Maroudas A., Stockwell R.A., Nachemson A., Urban J. (1975). Factors involved in the nutrition of the human lumbar intervertebral disc: Cellularity and diffusion of glucose in vitro. J. Anat..

[8] Ohshima H., Urban J.P. (1992). The effect of lactate and pH on proteoglycan and protein synthesis rates in the intervertebral disc. Spine.

[9] Repanti M., Korovessis P.G., Stamatakis M.V., Spastris P., Kosti P. (1998). Evolution of disc degeneration in lumbar spine: A comparative histological study between herniated and postmortem retrieved disc specimens. J. Spinal Disord..

[10] Bibby S.R., Fairbank J.C., Urban M.R., Urban J.P. (2002). Cell viability in scoliotic discs in relation to disc deformity and nutrient levels. Spine.

[11] Rajpurohit R., Risbud M.V., Ducheyne P., Vresilovic E.J., Shapiro I.M. (2002). Phenotypic characteristics of the nucleus pulposus: Expression of hypoxia inducing factor-1, glucose transporter-1 and MMP-2. Cell Tissue Res..

[12] Seagroves T.N., Ryan H.E., Lu H., Wouters B.G., Knapp M., Thibault P., Laderoute K., Johnson R.S. (2001). Transcription factor HIF-1 is a necessary mediator of the pasteur effect in mammalian cells. Mol. Cell. Biol..

[13] Epstein A.C., Gleadle J.M., McNeill L.A., Hewitson K.S., O’Rourke J., Mole D.R., Mukherji M., Metzen E., Wilson M.I., Dhanda A. (2001). C. elegans EGL-9 and mammalian homologs define a family of dioxygenases that regulate HIF by prolyl hydroxylation. Cell.

[14] Maxwell P.H., Wiesener M.S., Chang G.W., Clifford S.C., Vaux E.C., Cockman M.E., Wykoff C.C., Pugh C.W., Maher E.R., Ratcliffe P.J. (1999). The tumour suppressor protein VHL targets hypoxia-inducible factors for oxygen-dependent proteolysis. Nature.

[15] Mahon P.C., Hirota K., Semenza G.L. (2001). FIH-1: A novel protein that interacts with HIF-1alpha and VHL to mediate repression of HIF-1 transcriptional activity. Genes Dev..

[16] Kim J.W., Jeon N., Shin D.E., Lee S.Y., Kim M., Han D.H., Shin J.Y., Lee S. (2021). Regeneration in Spinal Disease: Therapeutic Role of Hypoxia-Inducible Factor-1 Alpha in Regeneration of Degenerative Intervertebral Disc. Int. J. Mol. Sci..

[17] Lando D., Peet D.J., Gorman J.J., Whelan D.A., Whitelaw M.L., Bruick R.K. (2002). FIH-1 is an asparaginyl hydroxylase enzyme that regulates the transcriptional activity of hypoxia-inducible factor. Genes Dev..

[18] Chen S., Fang X.Q., Wang Q., Wang S.W., Hu Z.J., Zhou Z.J., Xu W.B., Wang J.Y., Qin A., Fan S.W. (2016). PHD/HIF-1 upregulates CA12 to protect against degenerative disc disease: A human sample, in vitro and ex vivo study. Lab. Investig..

[19] Fujita N., Hirose Y., Tran C.M., Chiba K., Miyamoto T., Toyama Y., Shapiro I.M., Risbud M.V. (2014). HIF-1-PHD2 axis controls expression of syndecan 4 in nucleus pulposus cells. FASEB J..

[20] Zhang H., La Marca F., Hollister S.J., Goldstein S.A., Lin C.Y. (2009). Developing consistently reproducible intervertebral disc degeneration at rat caudal spine by using needle puncture. J. Neurosurg. Spine.

[21] Zou F., Jiang J., Lu F., Ma X., Xia X., Wang L., Wang H. (2013). Efficacy of intradiscal hepatocyte growth factor injection for the treatment of intervertebral disc degeneration. Mol. Med. Rep..

[22] Zhou X., Li J., Teng J., Liu Y., Zhang D., Liu L., Zhang W. (2021). microRNA-155-3p attenuates intervertebral disc degeneration via inhibition of KDM3A and HIF1α. Inflamm. Res..

[23] Guillemin K., Krasnow M.A. (1997). The hypoxic response: Huffing and HIFing. Cell.

[24] Jiang B.H., Zheng J.Z., Leung S.W., Roe R., Semenza G.L. (1997). Transactivation and inhibitory domains of hypoxia-inducible factor 1alpha. Modulation of transcriptional activity by oxygen tension. J. Biol. Chem..

[25] Hu C.J., Sataur A., Wang L., Chen H., Simon M.C. (2007). The N-terminal transactivation domain confers target gene specificity of hypoxia-inducible factors HIF-1alpha and HIF-2alpha. Mol. Biol. Cell.

[26] Luo Y., Jiang C., Belanger A.J., Akita G.Y., Wadsworth S.C., Gregory R.J., Vincent K.A. (2006). A constitutively active hypoxia-inducible factor-1alpha/VP16 hybrid factor activates expression of the human B-type natriuretic peptide gene. Mol. Pharmacol..

[27] Patel T.H., Kimura H., Weiss C.R., Semenza G.L., Hofmann L.V. (2005). Constitutively active HIF-1alpha improves perfusion and arterial remodeling in an endovascular model of limb ischemia. Cardiovasc. Res..

[28] Roberts S., Caterson B., Menage J., Evans E.H., Jaffray D.C., Eisenstein S.M. (2000). Matrix metalloproteinases and aggrecanase: Their role in disorders of the human intervertebral disc. Spine.

[29] Boos N., Nerlich A.G., Wiest I., von der Mark K., Aebi M. (1997). Immunolocalization of type X collagen in human lumbar intervertebral discs during ageing and degeneration. Histochem. Cell Biol..

[30] Inkinen R.I., Lammi M.J., Lehmonen S., Puustjärvi K., Kääpä E., Tammi M.I. (1998). Relative increase of biglycan and decorin and altered chondroitin sulfate epitopes in the degenerating human intervertebral disc. J. Rheumatol..

[31] Le Maitre C.L., Freemont A.J., Hoyland J.A. (2004). Localization of degradative enzymes and their inhibitors in the degenerate human intervertebral disc. J. Pathol..

[32] Sive J.I., Baird P., Jeziorsk M., Watkins A., Hoyland J.A., Freemont A.J. (2002). Expression of chondrocyte markers by cells of normal and degenerate intervertebral discs. Mol. Pathol..

[33] Antoniou J., Steffen T., Nelson F., Winterbottom N., Hollander A.P., Poole R.A., Aebi M., Alini M. (1996). The human lumbar intervertebral disc: Evidence for changes in the biosynthesis and denaturation of the extracellular matrix with growth, maturation, ageing, and degeneration. J. Clin. Investig..

[34] Bell D.M., Leung K.K., Wheatley S.C., Ng L.J., Zhou S., Ling K.W., Sham M.H., Koopman P., Tam P.P., Cheah K.S. (1997). SOX9 directly regulates the type-II collagen gene. Nat. Genet..

[35] Huang W., Zhou X., Lefebvre V., de Crombrugghe B. (2000). Phosphorylation of SOX9 by cyclic AMP-dependent protein kinase A enhances SOX9’s ability to transactivate a Col2a1 chondrocyte-specific enhancer. Mol. Cell. Biol..

[36] Wegner M. (1999). From head to toes: The multiple facets of Sox proteins. Nucleic Acids Res..

[37] Park J.B., Chang H., Kim K.W. (2001). Expression of Fas ligand and apoptosis of disc cells in herniated lumbar disc tissue. Spine.

[38] Richardson S.M., Walker R.V., Parker S., Rhodes N.P., Hunt J.A., Freemont A.J., Hoyland J.A. (2006). Intervertebral disc cell-mediated mesenchymal stem cell differentiation. Stem Cells.

[39] Nerlich A.G., Boos N., Wiest I., Aebi M. (1998). Immunolocalization of major interstitial collagen types in human lumbar intervertebral discs of various ages. Virchows Arch..

[40] Singleton R.S., Trudgian D.C., Fischer R., Kessler B.M., Ratcliffe P.J., Cockman M.E. (2011). Quantitative mass spectrometry reveals dynamics of factor-inhibiting hypoxia-inducible factor-catalyzed hydroxylation. J. Biol. Chem..

[41] Li Y., Zhang D., Wang X., Yao X., Ye C., Zhang S., Wang H., Chang C., Xia H., Wang Y.C. (2015). Hypoxia-inducible miR-182 enhances HIF1α signaling via targeting PHD2 and FIH1 in prostate cancer. Sci. Rep..

[42] Pfirrmann C.W., Metzdorf A., Zanetti M., Hodler J., Boos N. (2001). Magnetic resonance classification of lumbar intervertebral disc degeneration. Spine.

[43] Hsieh A.H., Hwang D., Ryan D.A., Freeman A.K., Kim H. (2009). Degenerative anular changes induced by puncture are associated with insufficiency of disc biomechanical function. Spine.

[44] Issy A.C., Castania V., Castania M., Salmon C.E., Nogueira-Barbosa M.H., Bel E.D., Defino H.L. (2013). Experimental model of intervertebral disc degeneration by needle puncture in Wistar rats. Braz. J. Med. Biol. Res..

[45] Nakayama E., Matsumoto T., Kazama T., Kano K., Tokuhashi Y. (2017). Transplantation of dedifferentiation fat cells promotes intervertebral disc regeneration in a rat intervertebral disc degeneration model. Biochem. Biophys. Res. Commun..

[46] Sakai D., Mochida J., Iwashina T., Hiyama A., Omi H., Imai M., Nakai T., Ando K., Hotta T. (2006). Regenerative effects of transplanting mesenchymal stem cells embedded in atelocollagen to the degenerated intervertebral disc. Biomaterials.

[47] Masuda K., Aota Y., Muehleman C., Imai Y., Okuma M., Thonar E.J., Andersson G.B., An H.S. (2005). A novel rabbit model of mild, reproducible disc degeneration by an anulus needle puncture: Correlation between the degree of disc injury and radiological and histological appearances of disc degeneration. Spine.

